# An Integrated Psychosocial Model of Relatives' Decision About Deceased Organ Donation (IMROD): Joining Pieces of the Puzzle

**DOI:** 10.3389/fpsyg.2018.00408

**Published:** 2018-04-10

**Authors:** Jorge S. López, Maria Soria-Oliver, Begoña Aramayona, Rubén García-Sánchez, José M. Martínez, María J. Martín

**Affiliations:** ^1^Departamento de Psicología y Pedagogía, Universidad Pública de Navarra, Pamplona, Spain; ^2^Departamento de Psicología Social y Metodología, Universidad Autónoma de Madrid, Madrid, Spain; ^3^Facultad de Ciencias de la Salud, UNIR-Universidad Internacional de la Rioja, Logroño, Spain

**Keywords:** organ donation, family consent, family decision making, conceptual analysis, psycho-social model, decision making under stress, altruism and prosocial behavior

## Abstract

Organ transplantation remains currently limited because the demand for organs far exceeds the supply. Though organ procurement is a complex process involving social, organizational, and clinical factors, one of the most relevant limitations of organ availability is family refusal to donate organs of a deceased relative. In the past decades, a remarkable corpus of evidence about the factors conditioning relatives' consent has been generated. However, research in the field has been carried out mainly by means of merely empirical approaches, and only partial attempts have been made to integrate the existing empirical evidence within conceptual and theoretically based frameworks. Accordingly, this work articulates the proposal of an Integrated Psychosocial Model of Relatives' Organ Donation (IMROD) which offers a systematic view of the factors and psychosocial processes involved in family decision and their interrelations. Relatives' experience is conceptualized as a decision process about the possibility of vicariously performing an altruistic behavior that takes place under one of the most stressful experiences of one's lifetime and in the context of interaction with different healthcare professionals. Drawing on this, in the proposed model, the influence of the implied factors and their interrelations/interactions are structured and interpreted according to their theoretically based relation with processes like rational/heuristic decision-making, uncertainty, stress, bereavement, emotional reactions, sense of reciprocity, sense of freedom to decide, and attitudes/intentions toward one's own and the deceased's organ donation. Our model also develops a processual perspective and suggests different decisional scenarios that may be reached as a result of the combinations of the considered factors. Each of these scenarios may imply different balances between factors that enhance or hinder donation, such as different levels of uncertainty and potential decisional conflict. Throughout our work, current controversial or inconsistent results are discussed and interpreted on the basis of the relationships that are posited in the proposed model. Finally, we suggest that the structure of the relationships and interactions contained in our model can be used by future research to guide the formulation of hypotheses and the interpretation of results. In this sense, specific guidelines and research questions are also proposed.

## Introduction

Organ transplantation is an established and cost effective medical procedure for end-stage organ failure. Whereas, organ transplantation has attained an appropriate level of development in terms of medico-surgical procedures, it remains limited by the gap between organ availability and the number of patients who would potentially benefit from receiving a transplant (Council of Europe O. N. T., 2016).

Currently, deceased organ donation is largely the most important source of human organs. Deceased organ donation rates are therefore a critical factor for organ transplantation. They vary widely across national contexts and are result of a complex process that includes, among others, social, economic, legal, and organizational factors (Council of Europe O. N. T., 2016). However, a key factor in the process of organ procurement has to do with the role of bereaved relatives when consent for donation from a deceased loved one is requested. Legislation in western countries regarding deceased organ donation consent includes both the so-called *opt-in* and *opt-out* systems. Opt-in systems require voluntary and explicit consent to proceed to donation and otherwise prescribe that the next-of-kin be consulted to determine the deceased's preferences about organ donation. Opt-out systems, in contrast, assume consent to donate in the absence of expressed objection. In any case, regardless of the existing legislation, real donation practices in the majority of countries (including leading donation countries like Spain, Portugal, the UK, or the USA) require family consent as a prerequisite for deceased organ donation (Rosenblum et al., 2012). Family refusal thus constitutes a potential limitation to organ procurement and, in fact, decreased organ availability in a relevant percentage of cases. Refusal rates in Spain in 2015 remained around 15%, but they contrast with the rate of Italy (30.3%) or the UK (34.2%) (Council of Europe O. N. T., 2016). USA organ procurement organization reported a wide range of refusals that range from 6.4 to 44.9% (UNOS, 2017).

There is currently a relevant body of literature focused specifically on the empirical study of the experience of relatives facing organ donation and the analysis of the factors influencing their decision. There are also excellent recent reviews in this field. This is the case of the works of: Chandler et al. (2017), which provides guidelines for “effective” requesting; Ralph et al. (2014), dedicated to qualitative studies; Walker et al. (2013), including both qualitative and quantitative empirical works; Siminoff et al. (2013), who used a global point of view; de Groot et al. (2012), focused on empirical, theoretical, and practical studies; and Simpkin et al. (2009), centered on the evidence about modifiable factors influencing relatives' decisions.

A global view of the cited works shows that family decision is a complex and dynamic process influenced by factors referring to different actors, levels, and temporal moments. The general relationship of some specific factors with family decision appears well grounded on the basis of empirical results. These factors are related to different aspects that coalesce in family decision processes, like the circumstances of death, the characteristics and dynamics of the deceased's family, the relatives' knowledge of the deceased's wishes about donation, the relatives' own attitudes toward organ donation and health-staff care, and information and organ donation request procedures. In any event, the above-mentioned works also reveal that little attention has been paid to clarify how these factors are articulated and interact from a global viewpoint. In addition, they show that results referring to some specific factors are inconsistent and need adequate conceptualization and explanation. This outcome is conditioned by the fact that most of the studies on family decision processes used a strongly empirical approach, without explicit reference to theoretical frameworks that could have served to elucidate and structure the factors' influence and interrelation. It is also explained by the fact that empirical works—especially in the case of quantitative studies—have focused almost exclusively on the bivariate or multivariate relation between family decision, on the one hand, and specific factors, on the other, paying little attention to the interactive relationships between the involved factors.

However, some relevant attempts have been made to conceptualize the experience of bereaved relatives from a theoretical viewpoint. In their earlier works, Pearson et al. (1995) and Pelletier (1992a,b) conceptualized the family process in terms of the stress coping model of Lazarus and Folkman. Radecki and Jaccard (1997) interpreted the existing empirical evidence on organ donation on the basis of Fishbein and Ajzen's attitude-behavior model and proposed a global view of the psychological processes involved in family consent. However, their model was mostly focused on the analysis of relatives' beliefs about next-of-kin donation and paid scarce attention to other relevant concurrent factors. Sque, Payne and collaborators carried out various empirical studies (Sque and Payne, 1996; Sque et al., 2005, 2006; Long et al., 2008), taking as reference decision-making frameworks, grief rationalization processes, and proposing a dissonant loss model (Sque and Payne, 1996). The dissonant loss model offers a comprehensive view of family decision process (see below) but does not articulate the influence on family decision of factors like health-staff interventions. Various empirical works have also taken as reference different dimensions of the grieving process and its interrelation with families' donation decision (Steed and Wager, 1998; Cleiren and Zoelen, 2002; Bellali and Papadatou, 2006; Merchant et al., 2008). However, they do not offer a global view of the factors that coalesce in family decision. Bellali and Papadatou (2007), by means of a qualitative study, generated a “model of decision-making in organ donation” which provided relevant insights into formal features of family decision process (see below). Nevertheless, like Sque and Payne's model, it does not articulate the way in which different concurrent factors influence relatives' decision. Robbins et al. (2001), conceptualized family decision-making by means of the transtheorical model of behavior change (TTM). Their approach was focused on relatives' decisional balance when donation was first requested and suggests interventions that may be more adequate as a function of family decisional stage. However, like other models, their work does not provide a global view of family experience. López, Martínez and collaborators performed different empirical studies (Martinez et al., 2001; López et al., 2008) taking as reference psychosocial frameworks like decision-making, persuasion, altruism, bereavement process, stress, and coping. In any case, their work, needs further structuring and specification. Finally, some recent studies have proposed global views of the factors involved in family decision. This is the case of the model of factors influencing decision process proposed by de Groot et al. (2016) and the thematic schema formulated by Ralph et al. (2014). However, these models also need further structuring in order to be applied operatively to family consent research. However, as mentioned, donation literature still lacks the proposal of a global and articulated framework that structures the different factors involved in the family consent process.

Taking the above into account, we believe that the existing knowledge about family decision would greatly benefit from the elaboration of a global framework that could integrate the results and clarify unclear, contradictory findings. We also think that it may be useful to ground this framework in contrasted theoretical proposals generated from basic and applied psychological research. Consequently, the objective of the present work is to build a global model of family decision about deceased organ donation based on the existing evidence and psychosocial referents. This model aims: firstly, to provide a structured and systematic overview of the factors related to family decision and their expected relationship with family consent; secondly, to specify the expected relationships and interactions between the factors involved in family decision processes; thirdly, to provide a processual view of the articulation of the considered factors within the different phases of deceased relatives' experience.

## Psychosocial grounding of family decision

Family consent about organ donation includes diverse processes that may be conceptualized through psychosocial theoretical frameworks. Relatives, who may have previously developed their own attitudes about organ donation, have to cope with a stressful situation in which grief for a loved one merges with the requirement of a specific decision-making process. This decision-making process is focused on the opportunity to vicariously perform an altruistic behavior in which one of the main criterion is the evocation of the deceased's wishes and expressions about his/her own organ donation. In this situation, family members must cope with the balance between their own beliefs and attitudes about organ donation and the evoked wishes of the deceased relative. In addition, this situation takes place through the interaction with different healthcare professionals, whose behavior, caregiving, and way of presenting and requesting donation may influence the relatives' feelings during the grieving process as well as their contextual beliefs and behavior about donating. Departing from such a concise description, some relevant psychosocial constructs emerge as potential contributors to the explanation of family decision. Among them, without being exhaustive, the following are worth mentioning:

### Decision-making under stress

The development of the so called dual-process approach has shown that human cognitive processing can take place by means of different operating principles and outcomes (see Sherman et al., 2014, for a general view). In this sense, decision-making may be strategic and calculated in certain conditions. But decisions can also be based on heuristics, biases, and other “non-rational” or intuitive tendencies (see Gigerenzer et al., 1999; Hertwig and Hoffrage, 2013). Moreover, according to recent refinements of the dual process accounts of cognition, the two procedures may also be combined in different ways as a function of personal and contextual factors (Ferguson et al., 2014). The study of decision-making under stress shows that high-stress situations may lead to higher reliance on lower level automatic response tendencies and to a decrease of controlled cognitive processes (Starcke and Brand, 2012). According to stress coping proposals (see Frydenberg, 2014, for a review), taking as reference some specific heuristics to guide decision-making may act as a clear decision referent that reduces the ambiguity of the situation, decreases the number of demands and, in consequence, reduces the high tension generated by the situation. As mentioned, some specific studies have approached relatives' organ donation on the basis of decision-making processes. Both empirical outcomes and the resulting conceptualization are highly concordant with the aforementioned theoretical proposal. In this sense, Sque and Payne (1996) showed that relatives experienced a complex bereavement and decision-making process that was characterized by a sense of uncertainty and psychological inconsistency, and they specified the sequential phases and conflicts that relatives face. In this sense, they proposed that relatives may use both rationalistic and emotion-based strategies. Bellali and Papadatou (2007) showed that relatives' decision-making process takes place at different parallel and interactive levels: at an individual/personal level and at an interpersonal level. They showed that decisions could take place either instantaneously or through a rational, stepwise decision-making process. Finally, López et al. (2008) found that relatives' decisions may be guided by the interaction of two main heuristics: the explicit or inferred will of the deceased and family attitudes to organ donation and transplant. All these mentioned evidences that clarify potential decision rules used by relatives and that analyze the factors that are operating may be useful tools to understand the family's experience.

### Altruism and prosocial behavior

The study of the factors conditioning prosocial behavior has a long tradition within social psychology. In any case, giving consent for organ donation of a deceased relative represents a particular case of prosocial behavior, which takes place in circumstances that are different from those considered in common research paradigms. The most important difference lies in the fact that prosocial behavior is required in the context of a situation that implies one's own extreme suffering. Considering this conditioning, the recently developed concept of “altruism born of suffering” (Staub, 2003, 2005) can be used as an excellent tool to understand families' experience. Using this concept, Vollhardt (2009) proposed a specific model that structures the motivational processes that may lead persons to behave altruistically in painful situations. In this sense, helping others would improve coping and post-traumatic growth by relieving the negative affect related to one's suffering, increasing self-efficacy, enhancing social integration/reward, and regaining meaning. Specific situational demands and norms may contribute to the emergence of helping behavior, such as required helpfulness in situations of need and the existence of the reciprocity norm of helping. Additionally, positive affect and common categorization with the potential aim of helping would lead to increased empathy and perspective-taking with others in need. Following Kuhl (1987) and Vollhardt (2009) also proposed some volitional process that may facilitate or hinder motivations for altruism born of suffering and that can be subject of intervention: selective attention, encoding control, emotional control, motivational control, and environmental control. Additionally, some contributions from research on prosocial behavior that could contribute to understanding donation decision should be mentioned: first, the positive link between empathy toward victims and altruistic behavior largely shown in the literature (Hoffman, 2008); the conceptualization of anger as an equity-restoring tool that may hinder prosocial behavior depending on its target (van Doorn et al., 2014); and the role of processes such as reciprocity, diffusion of responsibility, and the self-perception of the capacity to help, which are considered in traditional altruism literature (Batson and Powell, 2003; Penner et al., 2005).

### Grief and bereavement

The loss of a loved one leads to the development of grief, an extreme but normal variant of sadness that may evolve in different ways. Research in this area has questioned the former assumption of “grief work”—the idea that all grieving must go through a necessary series of stages after which the individual has to let go of the deceased person (Stroebe et al., 2001). Instead, recent complex models propose that an adequate approach to understanding grief should examine interactions between attachment style, type of relationship with the deceased, and coping strategies (Power and Dalgleish, 2008; Stroebe and Schut, 2010). In this sense, grief experience can run a number of possible courses depending on cultural and family pressure, individuals' developmental history, the nature of the relationship with the deceased, type and suddenness of death, and quality of support from significant others (Parkes and Prigerson, 2013). These kinds of grieving models offer different cues that may help to better understand bereaved relatives receiving a donation request. Power and Dalgleish (2008) clarify that loss of a loved one leads not only to the emotion of sadness, but frequently also to anger. In this sense, the expression of such ambivalence may be coped with in different ways according to personal and contextual factors: anger is more likely to occur when death is sudden or unexpected, and may be directed at the deceased, but also at others who caused the loss or did not do enough to prevent it. As mentioned, the factors enhancing anger may hinder the decision to perform prosocial behavior, especially if those involved in the organ donation request or those who will generically benefit from donation are perceived as part of the anger target. On another hand, as different studies have suggested (Sque and Payne, 1996; Martinez et al., 2001), emotional reactions that indicate that family members cannot process the fact of the loved one's death may hinder the possibility of even considering organ donation.

## An integrated model of relatives' organ donation (IMROD)

**Figure 1:**
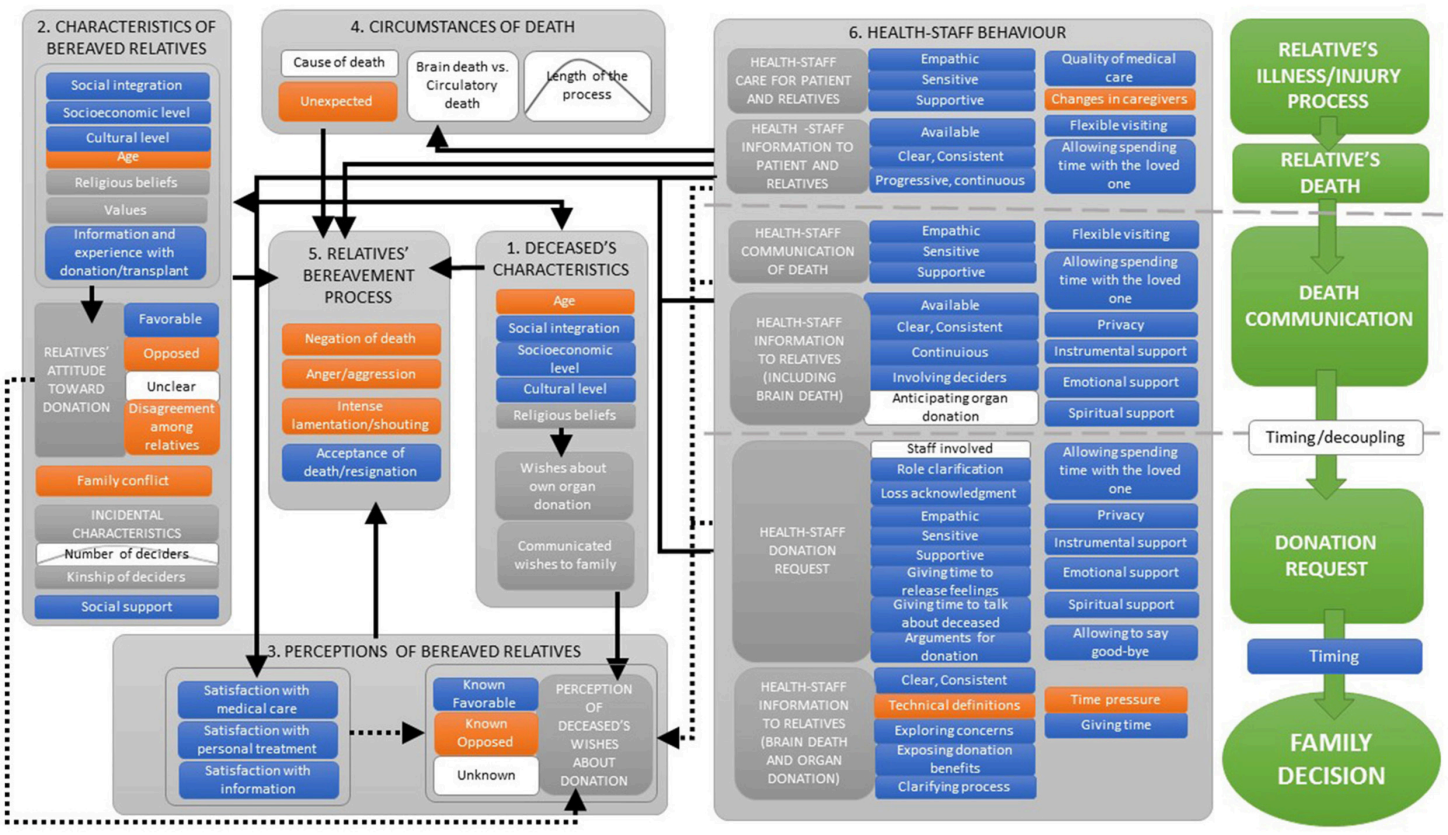
Integrated Model of Relatives' Organ Donation (IMROD): Included factors, relationship with family consent and general interrelationships between factors. 
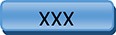
 Direct/positive relationship with family consent; 
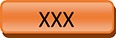
 Inverse/negative relationship with family consent; 
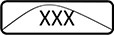
 Inverted U relationship with family consent; 
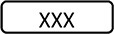
 Relationship depends mainly on other underlying factors; 
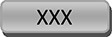
 Relationship with family consent varies across categories; 
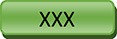
 Family experience process; 
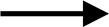
 Conceptually/empirically well-grounded relationships; 
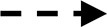
 Suggested innovative relationships.

**Figure 2:**
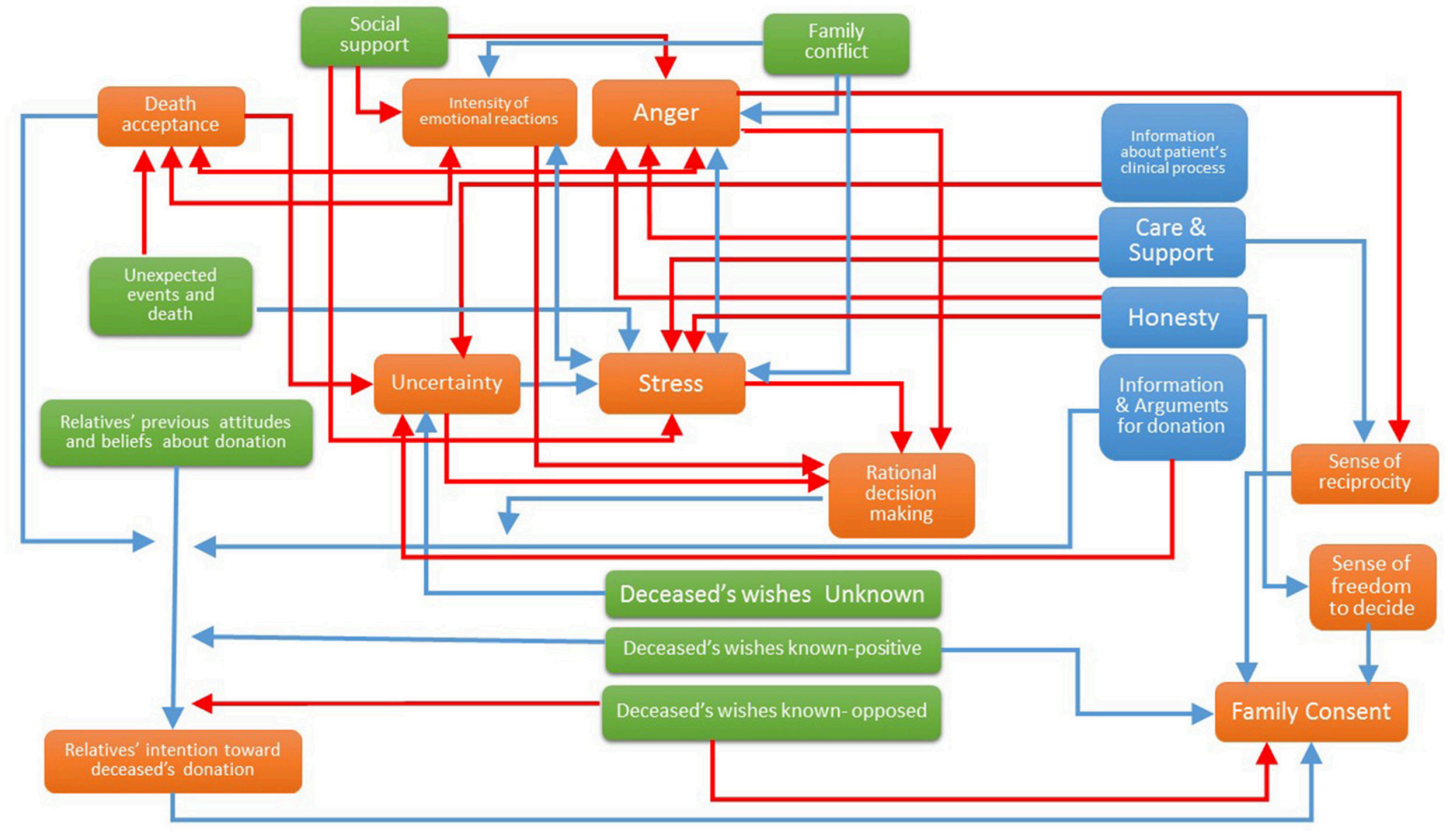
Integrated Model of Relatives' Organ Donation (IMROD): Perceptions/resources provided by health-staff intervention to relatives, Psychological processes experienced by relatives, Concurrent factors and their interrelationships. 
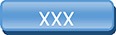
 Perceptions/resources provided by health-staff intervention to relatives; 
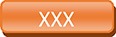
 Psychological processes experienced by relatives; 
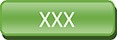
 Concurrent factors; 
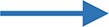
 Positive relationships; 
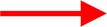
 Negative/inhibitory relationships.

**Figure 3:**
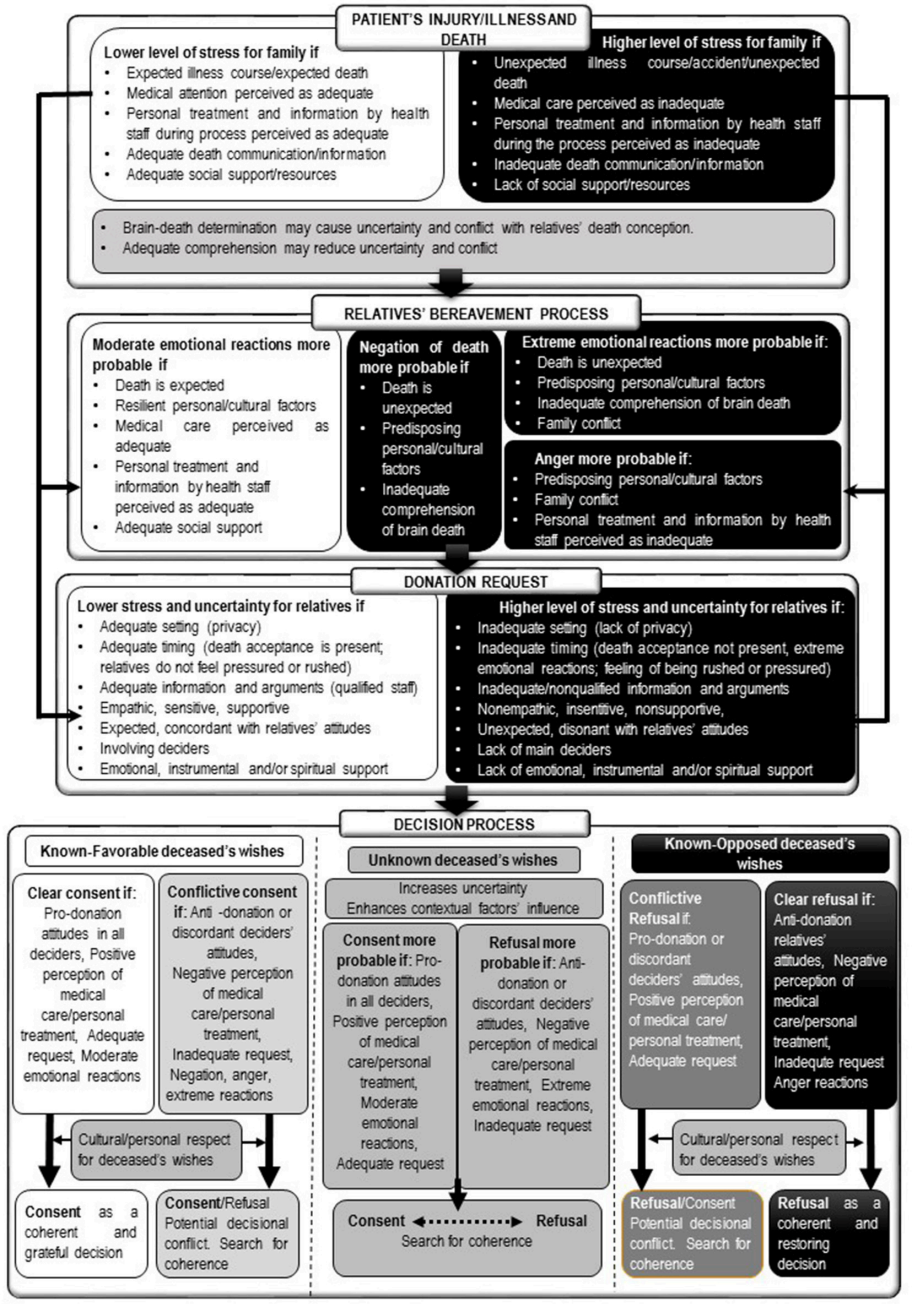
Processual view of family experience and family decision.

On the basis of the above-mentioned psychosocial frameworks and the existing literature on family decision-making regarding organ donation, an Integrated Model of Relatives' Organ Donation (IMROD) is proposed. IMROD is summarized in Figures 1–**3**. Figure 1 offers an exhaustive view of the measurable factors that may be related to family decision and their interrelations. Figure 2 summarizes which psychological processes are involved in family decision and represents their relationships with concurrent/contextual factors and health-staff interventions. Figure 3 offers a processual view of family experience and the role of the most relevant factors. In the following sections, we will explain the proposed model, detailing the included factors, their expected relationship with family decision, the expected interrelations and interactions between factors, and summarizing the processual view of family decision.

### Factors related to family-decision process, interrelationships and interactions

#### The deceased's characteristics

The deceased's characteristics may influence family decision in different ways. On the one hand, some sociodemographic variables are associated with differential attitudes about organ donation and may be related to the deceased's expressed wishes about donation. On the other hand, some of the deceased's characteristics may be related to the deciders' own characteristics and may condition the circumstances of the decision process. In this sense, on the basis of opinion polls, deceased people with a higher socioeconomic level, higher educational level, and adequate social integration may have expressed positive wishes to a greater extent (Ashkenazi et al., 2005; Mocan and Tekin, 2007; Scandroglio et al., 2011). The opposite should be inferred in the case of lower socioeconomic or educational level and lack of social integration. The deceased's age may condition family decision in a more complex way. As a general rule, age may have an inverse relationship with family consent, but some details should be underlined. In this sense, special conditions coalesce when the deceased is a baby or a child, as the grieving process involves a particularly emotional impact and, in almost all cases, donation would not have been discussed. The search of meaning for the loss and empathy toward other children may positively influence family decision (Ralph et al., 2014). In fact, in several studies, families of young children have shown greater willingness to donate than families of older children (Walker et al., 1990; Morris et al., 1992) or of adults (Pike et al., 1991). However, such results are not consistent across all studies (Pottecher et al., 1993). In relation to young adults, as in the case of children, the emotional impact of unexpected death on relatives also merges with the need to find meaning for the loss and, in this case, with a higher probability of expressed willingness to donate. In the case of older adults, expressed negative wishes are more probable, also according to opinion polls. Additionally, relatives may (often inappropriately) anticipate the potential non-utility of the deceased's organs for donation, thus hindering their self-perception of the capacity to help, and declining donation. In this sense, several studies evidence the expected lower donation rates in families of older adults when compared with younger groups (Rodrigue et al., 2006; van Leiden et al., 2010; Weiss et al., 2014). Religious beliefs also reveal complex relationships with the expression of willingness to donate. All major world religions approve of donation, but subgroups with a religious tradition may disagree (Chandler et al., 2017). Public surveys have also revealed a better disposition to donate in non-religious individuals and subgroups with moderate religious implication when compared with sectors with high religious implication (Scandroglio et al., 2011; López et al., 2012). Finally, the deceased's wishes about donation and their communication to the family play a central role in family decision. In fact, the deceased's wishes (known or imagined) are the most powerful and consistent predictor of family decision (Walker et al., 2013; Chandler et al., 2017). However, in the absence of the deceased's formal statement, the deceased's “real” wishes cannot be directly known. Consequently, the relatives' perception of the loved one's wishes becomes the adequate indicator that will be considered in our model in a further section.

#### Characteristics of the bereaved relatives

The family's characteristics, beliefs, and behavior also act as factors that influence the next-of-kin's decision through different processes. As a relevant basis, it has to be borne in mind that relatives tend to bias their decision about the deceased's organ donation toward their own attitudes on the issue, especially when the deceased's wishes are unknown (Sque and Payne, 1996; DeJong et al., 1998; López et al., 2008). As mentioned previously, specific sociodemographic groups may have different attitudes toward organ donation, which may condition their decision about organ donation of the deceased. In this sense, consent rates are lower in families with low socioeconomic level, low cultural level, and belonging to minority ethnic groups (de Groot et al., 2012; Chandler et al., 2017). Lack of information, religious fears, distrust of medical staff, and communication difficulties have been related to the negative disposition of these populations (Radecki and Jaccard, 1997). These difficulties may be critical when relatives must cope with the declaration of brain death and its explanation by the health staff (see below). Likewise, older relatives are expected to show a worse disposition toward donation. Relatives' religious beliefs, such as specific concerns related to brain death, desecration of the body, afterlife, and funerary rites, may also influence decision about donation (Chandler et al., 2017). However, although spirituality and religion are often mentioned as reasons for differences in the willingness to donate in surveys, real relatives' refusal based on religion seems scarce (de Groot et al., 2012). Relatives' values regarding protection, integrity, and respect for the body could also prevent them from donating, although such concerns have been shown to be easier to resolve with adequate information than other refusal reasons (Martinez et al., 2001). The importance given to honoring the deceased's wishes may mediate the relationship between relatives' knowledge of the deceased's willingness to donate and family decision, as we will detail below.

Prior knowledge about organ donation/transplantation and/or personal experience of this process positively influences family members' decision (Frutos et al., 2005b; Walker et al., 2013). This circumstance increases the emergence of positive attitudes toward donation and makes it more probable for the family to expect donation request, thus reducing its potential stressful impact on the family. An expected donation request on the basis of adequate information may even give the family an opportunity to give a sense of purpose to the loss (Ralph et al., 2014). In the same way, the family's positive attitudes and beliefs make a positive decision more probable (Walker et al., 2013). Conflicting views or disagreement about donation among relatives may enhance decisional stress, favor the emergence of negative emotions, and consequently hinder donation (Ralph et al., 2014). Additionally, in conflicting situations, relatives who favor donation have been shown to give priority to reducing the stress that donation may cause in the relatives opposed to donation, even in spite of their own wishes (de Groot et al., 2012; Walker et al., 2013).

In relation to family dynamics, the existence of poor family relationships and family conflict may add additional psychological distress to the grieving process and favor the emergence of negative emotions, thus hindering the altruistic behavior of donation. Poor family relationships and family conflict are thus related to lower consent rates (Martinez et al., 2001; Rodrigue et al., 2008). Regarding the decision-making process, family composition may vary also according to different circumstances and may have important influence on consent. In relation to the number of involved relatives, an inverted-U pattern is expected. Isolated relatives may suffer greater distress and lack of advice/social support that may hinder the act of donating. On another hand, the participation of a large number of relatives and extended family as deciders may decrease the probability of positive consensus and make an adequate support and request by health staff more difficult (Rodrigue et al., 2008). The specific influence of the deciders' kinship on consent rates is controversial and probably depends on the interaction of other factors (Weiss et al., 2014). Parents, however, have shown better consent rates in different studies when compared with spouses or descendants (Schaub et al., 2013; Weiss et al., 2014). The presence of adequate social support for relatives may contribute to buffering psychological distress, favoring rational decision-making and making donation decisions more probable when favorable attitudes prevail.

#### Perceptions of the bereaved relatives

The relatives' perceptions of different aspects of the process are key factors to determine the final decision. As mentioned, the perception of the deceased's wishes about donation is the strongest predictor of family decision (Walker et al., 2013; Chandler et al., 2017). However, a detailed analysis of its role is required. According to decision-making frameworks, at a time of great stress such as this, it is especially useful for the family to have a clear decision referent or heuristic decision rule that reduces the ambiguity of the situation and decreases psychological distress (Starcke and Brand, 2012). In this sense, knowing that the deceased was in favor of or against donation gives the relatives a clear decision rule. However, as we will see in the processual view of the proposed model, such a criterion can be concordant or discordant with other factors that converge in the entire process—mainly, relatives' attitudes about donation and their perception of health-staff care—thus generating different decisional settings and different decision outcomes. However, the level of respect toward the deceased's wishes varies across individuals and cultures and may mediate the relationship between knowledge of the deceased‘s willingness to donate and family decision. In fact, the literature has shown great differences in the degree to which relatives follow the deceased's expressed willingness in different cultural contexts (Martinez et al., 2001; de Groot et al., 2016). Additionally, in the absence of a formal declaration, the deceased's willingness to donate is not necessarily remembered as an objective fact. On the contrary, it may be inferred on the basis of the relatives' recall and, as has been suggested (López et al., 2008), it may be conditioned by the influence of different concurrent factors. When the deceased's wishes are unknown, and no reference can be evoked by the relatives, decisional uncertainty increases and concurrent factors may become more relevant. In this sense, it is expected that the influence of concurrent factors on family decision may interact with the relatives' knowledge of the deceased's wishes: influence of concurrent factors may be stronger when the deceased's wishes are unknown and weaker when a clear positive or negative will is present.

Relatives' perception of the health-staff's behavior and interventions is also one of the most relevant predictors of family decision (Frutos et al., 2005a; Simpkin et al., 2009; de Groot et al., 2012; Chandler et al., 2017). Such perception has relevant implications in dimensions like the level of distress and uncertainty with which the family must cope, the emergence and/or modulation of the family's emotional reactions, and the emergence of a sense of reciprocity. Family perceptions may be based on a wide range of interventions that take place during the relative's illness/injury or death, upon donation request, and in donation decision processes. We will perform an in-depth analysis of the specific health-staff interventions that modulate family decision and the underlying psychological processes related to them in the section dedicated to health-staff's behavior.

#### Circumstances of death

Circumstances related to the cause and timing of death potentially impact family decision. Some studies found that consent rate may vary according to the specific cause of death (Pike et al., 1991; Gortmaker et al., 1998), but scarce attention has been paid to this factor in current literature. In this sense, the effect of the cause of death on relatives' decisions may be more adequately explained as a function of other factors. Among them, the degree to which the death of the loved one is unexpected may be related to higher psychological distress that could affect the relatives' bereavement process and decision about donation. Additionally, according to coping models, the length of the process may influence family decision following an inverted-U pattern. When the process that leads to the relative's death takes place in a short period of time, the surviving relatives' cognitive and emotional resources may be overwhelmed. On another hand, a prolonged stay in the hospital may undermine relatives' resources and also cause psychological distress. Both circumstances decrease the relatives' ability to cope with the loss and make the decision to donate more difficult. Differences in family decision between situations in which brain-stem death and circulatory death have occurred are also relevant. To date, brain-stem death has comprised the largest pool of potential donors but donations derived from circulatory death are steadily increasing (Council of Europe O. N. T., 2016). Brain-death declaration is based on the diagnosis and confirmation of the irreversible cessation of the functioning of the entire brain, including the brain stem (Shemie et al., 2014). Although brain-death determination is a well-established medical procedure, it disagrees with the common social conception of death, as respiratory and cardiac activity may continue, and the deceased may seem alive to the relatives. Consequently, relatives are often shocked and stunned by brain-death diagnosis and may even express disbelief in the validity of the diagnosis (de Groot et al., 2012). In this sense, an inadequate comprehension of brain death may seriously hinder death acceptance, increase uncertainty and stress, and prevent relatives from considering donation. However, family members have been shown to accept donation without understanding the procedures used to diagnose brain death and also still feeling unsure about when the moment of death occurred (Long et al., 2008; Walker et al., 2013). Acceptance of donation in such circumstances is possible when other factors of the process are favorable (i.e., positive and known willingness of the deceased, trust and good relations with health staff). Such evidence shows that, in some cases, family decision-making is not necessarily logical. As has been proposed in current decisional frameworks, it illustrates that heuristics and rational thinking may be combined in some decision processes (Ferguson et al., 2014). Circulatory-death diagnosis better matches the common population conception of death and may consequently imply less uncertainty for families. However, donation procedures linked to circulatory death involve a short time period and force the health staff to perform the donation request and the families to make their decision in reduced time settings, thus enhancing distress. As a global result, the difference between family donation rates in case of brain death and circulatory death are not consistent across the different studies. Both lower and higher family refusal rates for organ donation have been found in non-heart-beating vs. brain-dead donors (Barber et al., 2006; Andrés et al., 2009). However, one of the most recent and broader studies found no demonstrable differences between the two situations (Siminoff et al., 2017).

#### Relatives' bereavement process

The emotional and cognitive state of the relatives is decisive to configure the decision-making process about organ donation. As mentioned above, current research on the grieving process does not conceptualize it as a prefigured sequence of phases and stages to predict the course of bereavement (Parkes and Prigerson, 2013). In any case, some specific reactions may be clearly related to relatives' refusal. First, the family's lack of acceptance of the relative's death will turn the donation request into a particularly inappropriate and stressful event for the relatives, regardless of the objective fact of the loved one's death. Second, intense emotional reactions or reactions that indicate that the relatives' resources are overwhelmed may hinder their involvement in the decision process about donation and generate a refusal to avoid additional stressors. Third, angry reactions, especially if the family perceives that not enough was done to prevent the death, may highly conflict with the idea of donating. Anger may seriously hinder organ donation and could act as solid basis for refusal, especially if the health staff is included as part of the target of anger. In this situation, relatives may consider refusal as an adequate response to the inadequate care provided. However, although a relative's death is, in any case, one of the most stressing facts of the life course, more moderate emotional/cognitive reactions may allow deliberation of the possibility of donation. In this case, although structured rational decision-making may be somehow conditioned, and the use of heuristics may emerge, arguments about the benefits of donating may be considered, and family concerns could also be presented and discussed. In any case, rational decision-making may not lead to organ donation if clear and consolidated arguments or beliefs against donation are present. As anticipated, the family's emotional reactions may be conditioned not only by pre-existing factors like the individuals' developmental history or the relationship with the deceased, but also by contextual factors like the suddenness of the death, the quality of support, the perception of the medical care, and the existence of family conflict.

#### Health-staff's behavior

Care-givers' intervention is the most relevant modifiable factor of the donation process (Simpkin et al., 2009). In this sense, a wide range of actions are well established as good practices but some specific strategies remain controversial (Siminoff et al., 2013; Chandler et al., 2017). A previous analysis of the psychosocial process involved in family decision would be useful to allow a better understanding of the existing evidence and to enhance the interpretation of controversial results about the effects of health-staff interventions. These processes are also summarized in Figure 2. If we consider family consent as a decision-making process about the possibility of performing an altruistic behavior in the context of a stressful situation, the following health-staff interventions will contribute to increase the probability of donating by enhancing different interrelated psychological processes: (1) Interventions that reduce family stress. (2) Interventions that reduce relatives' uncertainty. (3) Interventions that facilitate the emergence of relatives' feelings of reciprocity toward the health-care system and potential donation recipients. (4) Interventions that promote the perception of donation as a free—as opposed to forced or manipulated—decision to perform an altruistic behavior. (5) Interventions that promote relatives' positive attitudes and/or intention to donate in the specific situation of the deceased relative's death. This group of interventions may include, on the one hand, interventions that maximize beliefs about the positive consequences of donation. On the other hand, it may include interventions that solve the existing concerns about donation, thus minimizing the perception of the negative consequences of donating. (6) Interventions that make rational decision-making more probable, with the exception of cases in which there are pre-existing prefigured and structured values or beliefs that oppose donation. Taking this into account, health-staff interventions can also be conceptualized according to the degree to which they provide relatives with some essential perceptions/resources (Figure 2): (1) Care, which may be related to the feeling that the health-staff's medical and personal interventions have tried to preserve the relative's and the family's physical and psychological well-being and may emerge through a broad variety of interventions. In this sense, care may be mainly related to stress reduction and the emergence of a sense of reciprocity. (2) Adequate information about the condition and diagnosis of the deceased, which may be related mainly to uncertainty reduction and, consequently, stress reduction. (3) Adequate information about organ donation, which should facilitate knowledge about the benefits of organ donation and solve potential concerns about it. Such interventions may influence by reducing uncertainty when making decisions about donation and by increasing relatives' positive attitude and/or intention toward organ donation of their loved one. (4) Honesty, which may be related to the health-staff behaviors that make the relatives feel they have not been pressured or manipulated beyond their own well-being in order to obtain consent. These interventions may help relatives feel that donation is a free altruistic decision.

Figure 1 summarizes the concrete interventions that, according to the existing evidence, are related to family decision process (Simpkin et al., 2009; de Groot et al., 2012; Walker et al., 2013; Chandler et al., 2017). Personal care for the patient's relatives that is perceived as empathic, sensitive, and supportive helps the family to cope with the highly stressful situation of the patient's illness or injury. When health staff is perceived as available, and information is given in a clear, consistent, progressive, and continuous way, relatives' uncertainty will decrease. Allowing the family to spend time with the loved one and offering flexible visiting hours may also enhance family well-being and reduce distress. On the contrary, changes in caregivers have been shown to decrease relatives' situational control and to generate distress. The perception that medical attention was adequate may reduce distress, avoid anger and negative emotions toward the health staff and the health-care system; it may also facilitate the emergence of a sense of reciprocity and altruistic feelings. Adequate death communication should be empathic, sensitive, and supportive. In the case of brain death, clear and unambiguous terms and explanations are especially relevant, and consent rates have been shown to be higher when enough time has been given to explain and discuss potential doubts (Chandler et al., 2017). However, technical language or profuse technical explanations, as well as discrepant information from different sources may overwhelm relatives and hinder donation (Ralph et al., 2014). Allowing them to spend time with the loved one, to say goodbye, ensuring privacy, and facilitating adequate instrumental, emotional, and, when required, spiritual support also helps families to cope with the loss and reduces distress.

In relation to donation request, some specific interventions are widely considered as good practices to enhance family consent, whereas others are already the subject of discussion. Within the first group, the following interventions are well established as good practices (Simpkin et al., 2009; de Groot et al., 2012; Siminoff et al., 2013; Walker et al., 2013; Chandler et al., 2017): ensuring a private location; participation of personnel with expertise in the field of organ donation and adequate training in effective requesting; sensitivity and compassion of the approach and the requestor; initiating the request in an empathic manner with an adequate introduction, role clarification, and an acknowledgment of the loss; performing confident, pro-donation approaches vs. nervous, guarded, or apologetic approaches; giving time to release feelings and talk about the deceased; avoiding questions that may tacitly encourage refusal to donate; developing a supportive, trust-based relationship with the family, which includes addressing concerns and questions with sensitivity; providing information and positive arguments about the benefits of donation; providing instrumental, emotional and, if required, spiritual support; ensuring that families do not to feel harassed or pressured; and giving families sufficient time for decision-making. Those practices integrate care for family well-being as an essential dimension of the request process and help to reduce the additional stress and uncertainty that may be caused by donation request. They also allow positive aspects of donation to be presented and potential concerns discussed. On the contrary, approaches that neglect family care, that are perceived by families as exclusively focused on organ donation or as manipulative may hinder donation decisions, as well as approaches that induce negative feelings (such as guilt or regret). Both approaches may undermine the feeling of the health staff's honesty and the conditions that allow a free altruistic behavior.

In any case, some practices have yielded inconsistent or contradictory results in empirical studies and may be analyzed in detail. In this sense, the optimal moment when donation may be raised cannot be directly inferred from the existing empirical results. A relevant number of studies have found that the practice of “decoupling” (separating the discussion of donation from the preceding notification of death) is related to higher consent rates (Simpkin et al., 2009; Chandler et al., 2017). However, several studies have failed to demonstrate such a relationship, and specific studies have even found better consent rates when donation was raised before the pronouncement of death (Siminoff et al., 2002; Nathan et al., 2003; Simpkin et al., 2009; Chandler et al., 2017). As different authors state (Rodrigue et al., 2008; Weiss et al., 2014) and also according to our model, the key factor of timing may not be determined by the fact that death declaration and donor request take place within a short time period; on the contrary, the critical factor is the degree to which relatives have accepted the deceased relative's death when organ donation request is performed, regardless of whether the formal declaration of brain death was made. In this sense, although decoupling is an accessible and widely used indicator within the literature, it may include under the same category situations that may diverge substantively in other critical variables (degree of death acceptance, emotional reactions, and stress level, among others). In this sense, practitioners should not take decoupling as the main and strict criterion for performing an adequate request; they should instead tactfully follow the family's emotional process in order to raise donation when the relative's death is seen by family as a definitive and irreversible fact.

Likewise, in relation to the health-staff's participation in donation request, the existing evidence does not allow establishing a general rule as to whether trained organ procurement personnel or health-care team members could be more effective to facilitate donation (Chandler et al., 2017). Additionally, donation rates in some studies have been shown to be higher when the family member and the person asking for consent have never met before (Rodrigue et al., 2006), but other studies have found an association between familiarity and donation rates (Rodrigue et al., 2008). From a theoretical point of view, when medical and personal care is perceived as adequate by relatives, health-care team members' request may have the advantage of higher levels of familiarity and the previously established trust of the relatives. Donation request, in this case, should also be less frequently perceived as interested or manipulative. Trained organ procurement staff, on another hand, may positively influence family consent as a result of their specific communication skills and their greater confidence when solving relatives' concerns; however they may be more strongly perceived as exclusively donation focused. The practice of combining the advantages of both requestors through what has been called “collaborative request” may be an effective practice, from a theoretical point of view. In collaborative request, members of the clinical team and organ procurement personnel jointly participate in donation request. Trust in the organ procurement personnel may be enhanced if clinical team members with a previous positive relation with the families promote their positive perception of the organ procurement personal. However, if medical and personal care was evaluated negatively by the families, organ procurement personnel may be more effective if they introduce themselves separately from the clinical team. In this sense, some studies have found greater donation rates when collaborative requests have been performed (Klieger et al., 1994; Gortmaker et al., 1998), although a recent randomized trial found no differences (ACRE Trial Collaborators, 2009).

### Processual view of relatives' experience and decision-making process

In order to complement the systematic structuring of the factors and psychological processes involved in family decision, Figure 3 offers a processual view of relatives' experience. We have tried to include within this processual view the evidence of the most relevant variables involved in the process. Additionally, we have tried to integrate the theoretical contributions of the previous works that have been considered as the most relevant (Sque and Payne, 1996; Radecki and Jaccard, 1997; López et al., 2008; Ralph et al., 2014; de Groot et al., 2016).

Our model structures the factors that may modulate the most relevant psychological processes that condition family decision-making across the different phases of family experience and the decision process: stress, uncertainty, emotional reactions, family likelihood to donate deceased organs, and rational/heuristic decision-making. As a result of the interaction between pre-existing and concurrent factors in different phases, we have posed different decisional settings that are classified according to the degree to which they may lead to donation decisions. Our central hypothesis is that relatives need to find coherence between the concurrent factors, their perception of the deceased's wishes about donation, and their own attitudes. When all factors converge in the same direction (consent/refusal), family decision may be predictable and consistent and may not generate decisional conflict, regardless of whether consent or refusal is adopted. When the concurrent factors and/or the relatives' own attitudes about organ donation enter into conflict with the deceased's wishes, family decision may be greatly mediated by the degree to which the relatives assume the deceased's wishes as a value to be respected. In this situation, decisional conflict may exist, and relatives may be induced to reduce dissonance and search for coherence by means of different secondary elaborations. If the value of honoring the deceased's wishes is highly salient, it will serve as the main referent for decision-making and will help to reduce potential dissonance between the final decision and other concurrent factors (e.g., relatives' own attitudes or perception of medical attention). When the deceased's wishes are unknown, decision-making is affected to a higher degree by uncertainty, as the heuristic of following the deceased's wishes is unavailable. In this situation, family decision may be an outcome of the confluence of their own attitudes, the concurrent factors, and the degree to which explanations and arguments about donation can be considered. Other heuristics may be used to guide decision-making, such as following a sense of reciprocity, thus giving a positive or negative response to donation request according to the positive or negative perception of the health-staff's care. However, if the relatives' emotional situation allows structured decision-making, they may be more permeable to information that enhances the benefits of donating and solves their potential concerns about it. Decisional conflict may, in any case, emerge if contrasting influences take place (e.g., relatives' positive attitude toward donation but negative perception of health care received or vice versa), and relatives may need to elaborate their perceptions to avoid dissonance.

## Implications for research

By means of the Integrated Psychosocial Model of Relatives' decision about Organ Donation (IMROD), we have provided a systematic view of the main factors influencing family consent and have also detailed which, in our view, are the most relevant psychological processes underlying relatives' decision. Our model also proposes a tentative structure of the relationships and interactions between the considered factors, thus suggesting different hypotheses that can be tested by empirical research.

In our view, our model shows that the application of a psychosocial conceptual analysis greatly enhances the interpretation of the existing empirical evidence about family decision. In this sense, we think that donation research, and especially quantitative studies, may greatly benefit from using a more structured and theoretically based approach. Such an approach could help the formulation of research hypotheses and guide the interpretation of results, especially those that seem contradictory or inconsistent. As we have suggested in different sections, unclear results across studies may be conditioned by the existence of potential interactions among factors. Some specific conditions or interventions may have different influences on family decision according to the degree to which other factors converge.

Some suggestions should be made to guide future research or even a detailed analysis of the existing data. First, donation research should not only be focused on the relationship between the selected factors and the family; it should also approach the analysis of interrelationships between predictors. In this sense, we suggest some specific research questions that should be tested empirically: How are family characteristics, circumstances of death, and health-staff intervention related to the family's bereavement process and emotional reactions? In the absence of a formal registration, how is the evocation of the deceased's wishes about donation conditioned by the circumstances of death and health-staff interventions? Second, empirical research in the field should carefully analyze potential interactions between some essential factors. Specifically: Does the influence of relatives' characteristics and emotional reactions and health-staff intervention have different predictive power on family decision when the deceased's wishes are unknown? Does the influence on family decision of donation request practices like decoupling or collaborative request vary as a function of factors like the family's emotional reactions or their perception of the quality of health-staff care?

Although our work has tried to elucidate the way in which different health-staff interventions could enhance the probability of family consent, the elaboration of specific guidelines for practitioners exceeds the scope of this paper. In any case, we hope that our proposals may help both empirical research and practice-focused works to develop specific suggestions and practices that increase family consent in an effective and grounded way.

## Author contributions

JL was on charge of work coordination and integration, so as final manuscript version. BA and RG-S were responsible for updating and summarizing the existing literature about family consent. MS-O reviewed and updated those psychosocial frameworks that may potentially ground the analysis of family decision. JM and MM worked on the integration of the empirical and theoretical contributions of literature within a global framework and the graphical representation of the proposed model. All authors reviewed the initial model draft and make contributions to its global articulation. They also reviewed manuscript final version.

### Conflict of interest statement

The authors declare that the research was conducted in the absence of any commercial or financial relationships that could be construed as a potential conflict of interest.

## References

[B1] ACRE Trial Collaborators (2009). Effect of “collaborative requesting” on consent rate for organ donation: randomised controlled trial (ACRE trial). Br. Med. J. 339:b3911 10.1136/bmj.b3911 19815583 PMC2759437

[B2] Andrés A. Morales E. Vázquez S. Cebrian M. P. Nuño E. Ortuño T.. (2009). Lower rate of family refusal for organ donation in non heart-beating versus brain-dead donors. Transplant. Proc. 41, 2304–2305. 10.1016/j.transproceed.2009.06.039 19715903

[B3] Ashkenazi T. Guttman N. Hornik J. (2005). Signing on the dotted line. Mark. Health Serv. 25, 19–24. 16001755

[B4] Barber K. Falvey S. Hamilton C. Collett D. Rudge C. (2006). Potential for organ donation in the United Kingdom: audit of intensive care records. BMJ 13, 1124–1127. 10.1136/bmj.38804.658183.55 PMC1459557 16641118

[B5] Batson C. D. Powell A. A. (2003). Altruism and prosocial behavior, in Handbook of Psychology: Personality and Social Psychology, Vol. 5, eds Millon T. Theodore Lerner M. J. (New York, NY: John Wiley and Sons), 463–484.

[B6] Bellali T. Papadatou D. (2006). Parental grief following the brain death of a child: does consent or refusal to organ donation affect their grief? Death Stud. 30, 883–917. 10.1080/07481180600923257 17024783

[B7] Bellali T. Papadatou D. (2007). The decision-making process of parents regarding organ donation of their brain dead child: a Greek study. Soc. Sci. Med. 64, 439–450. 10.1016/j.socscimed.2006.09.006 17064833

[B8] Chandler J. A. Connors M. Holland G. Shemie S. D. (2017). “Effective” requesting: a scoping review of the literature on asking families to consent to organ and tissue donation. Transplantation 101, S1–S16. 10.1097/TP.0000000000001695 28437367

[B9] Cleiren M. P. Van Zoelen A. A. (2002). Post-mortem organ donation and grief: a study of consent, refusal and well-being in bereavement. Death Stud. 26, 837–849. 10.1080/07481180290106607 12440423

[B10] Council of Europe and O. N. T (2016). Newsletter Transplant. International Figures on Organ Donation and Transplantation. Volume 21. Madrid: Council of Europe and Organización Nacional de Trasplantes.

[B11] de Groot J. van Hoek M. Hoedemaekers C. Hoitsma A. Schilderman H. Smeets W.. (2016). Request for organ donation without donor registration: a qualitative study of the perspectives of bereaved relatives. BMC Med. Ethics 17:38. 10.1186/s12910-016-0120-6 27401351 PMC4940748

[B12] de Groot J. Vernooij-Dassen M. Hoedemaekers C. Hoitsma A. Smeets W. van Leeuwen E. (2012). Decision making by relatives about brain death organ donation: an integrative review. Transplantation 93, 1196–1211. 10.1097/TP.0b013e318256a45f 23318303

[B13] DeJong W. Franz H. G. Wolfe S. M. Nathan H. Payne D. Reitsma W.. (1998). Requesting organ donation: an interview study of donor and nondonor families. Am. J. Crit. Care 7, 13–23. 9429679

[B14] Ferguson M. J. Mann T. C. Wojnowicz M. (2014). Rethinking duality: criticisms and ways forward. Invited chapter, in Dual-Process Theories of the Social Mind, eds Sherman J. W. Gawronski B. Trope Y. (New York, NY: Guilford Publications), 578–594.

[B15] Frutos M. A. Blanca M. J. Mansilla J. J. Rando B. Ruiz P. Guerrero F.. (2005a). Organ donation: a comparison of donating and nondonating families. Transplant. Proc. 37, 1557–1559. 10.1016/j.transproceed.2005.02.048 15866672

[B16] Frutos M. A. Blanca M. J. Ruiz P. Mansilla J. J. Seller G. (2005b). Multifactorial snowball effect in the reduction of refusals for organ procurement. Transplant. Proc. 37, 3646–3648. 10.1016/j.transproceed.2005.08.057 16386492

[B17] Frydenberg E. (2014). Coping research: historical background, links with emotion, and new research directions on adaptive processes. Aust. J. Psychol. 66, 82–92. 10.1111/ajpy.12051

[B18] Gigerenzer G. Todd P. M. ABC Research Group (1999). Simple Heuristics that Make Us Smart. New York, NY: Oxford University Press.

[B19] Gortmaker S. L. Beasley C. L. Sheehy E. Lucas B. A. Brigham L. E. Grenvik A.. (1998). Improving the request process to increase family consent for organ donation. J. Trans. Coordinat. 8, 210–217. 10205460 10.7182/prtr.1.8.4.2g64j1x161620765

[B20] Hertwig R. Hoffrage U. (eds.). (2013). Simple heuristics: the foundations of adaptive social behavior, in Simple Heuristics in a Social World (New York, NY: Oxford University Press), 3–36.

[B21] Hoffman M. L. (2008). Empathy and prosocial behavior, in Handbook of Emotions, eds Lewis M. Haviland-Jones J. M. Barrett L. F. (New York, NY: Guilford Press), 440–455.

[B22] Klieger J. Nelson K. Davis R. van Buren C. Davis K. Schmitz T. (1994). Analysis of factors influencing organ donation consent rates. J. Transpl. Coord. 4, 132–134.

[B23] Kuhl J. (1987). Action control: the maintenance of motivational states, in Motivation, Intention, and Volition, eds Halisch F. Kuhl J. (New York, NY: Springer), 279–291.

[B24] Long T. Sque M. Addington-Hall J. (2008). Conflict rationalisation: how family members cope with a diagnosis of brain stem death. Soc. Sci. Med. 67, 253–261. 10.1016/j.socscimed.2008.03.039 18442874

[B25] López J. S. Martín M. J. Scandroglio B. Martínez J. M. (2008). Family perception of the process of organ donation. Qualitative psychosocial analysis of the subjective interpretation of donor and nondonor families. Spanish J. Psychol. 11, 125–136. 10.1017/S1138741600004182 18630655

[B26] López J. S. Valentín M. O. Scandroglio B. Coll E. Martín M. J. Sagredo E.. (2012). Factors related to attitudes toward organ donation after death in the immigrant population in Spain. Clin. Transplant. 26, E210–E212. 10.1111/j.1399-0012.2011.01586.x 22283230

[B27] Martinez J. M. López J. S. Martín A. Martín M. J. Scandroglio B. Martín J. M. (2001). Organ donation and family decision-making within the Spanish donation system. Soc. Sci. Med. 53, 405–421. 10.1016/S0277-9536(00)00345-2 11459393

[B28] Merchant S. J. Yoshida E. M. Lee T. K. Richardson P. Karlsbjerg K. M. Cheung E. (2008). Exploring the psychological effects of deceased organ donation on the families of the organ donors. Clin. Transplant. 22, 341–347. 10.1111/j.1399-0012.2008.00790.x 18312444

[B29] Mocan N. Tekin E. (2007). The determinants of the willingness to donate an organ among young adults: evidence from the United States and the European Union. Soc. Sci. Med. 65, 2527–2538. 10.1016/j.socscimed.2007.07.004 17765372

[B30] Morris J. A. Wilcos T. R. Frist W. H. (1992). Pediatric organ donation: the paradox of organ shortage despite the remarkable willingness of families to donate. Pediatrics 89, 411–415. 1741213

[B31] Nathan H. M. Conrad S. L. Held P. J. McCullough K. P. Pietroski R. E. Siminoff L. A.. (2003). Organ donation in the United States. Am. J. Transplant. 3, 29–40. 10.1034/j.1600-6143.3.s4.4.x 12694048

[B32] Parkes C. M. Prigerson H. G. (2013). Bereavement: Studies of Grief in Adult Life. London, UK: Routledge.

[B33] Pearson I. Y. Bazeley P. Spencer-Plane T. Chapman J. R. Robertson P. (1995). A survey of families of brain dead patients: their experiences, attitudes to organ donation and transplantation. Anaesth. Intensive Care 23, 88–95. 7778754 10.1177/0310057X9502300116

[B34] Pelletier M. (1992a). The organ donor family members' perception of stressful situations during the organ donation experience. J. Adv. Nurs. 17, 90–97. 10.1111/j.1365-2648.1992.tb01822.x 1537995

[B35] Pelletier M. (1992b). Emotions experienced and coping strategies used by family members of organ donors. Can. J. Nurs. Res. 25, 63–73. 8118764

[B36] Penner L. A. Dovidio J. F. Piliavin J. A. Schroeder D. A. (2005). Prosocial behavior: multilevel perspectives. Annu. Rev. Psychol. 56, 365–392. 10.1146/annurev.psych.56.091103.070141 15709940

[B37] Pike R. E. Kahn D. Jacobson J. E. (1991). Demographic factors influencing consent for cadaver organ donation. South Afr. Med. J. 79, 264–267. 2011806

[B38] Pottecher T. Jacob F. Pain L. Simon S. Pivirotto M. L. (1993). Information des familles de donneur d'organes. Facteurs d'acceptation ou de refus du don. Resultats d'une enquete multicentrique. Ann. Franç. D'anesth. Réanim. 12, 478–482. 10.1016/s0750-7658(05)80995-5 8311354

[B39] Power M. Dalgleish T. (2008). Cognition and Emotion: From Order to Disorder. New York, NY: Psychology Press.

[B40] Radecki C. M. Jaccard J. (1997). Psychological aspects of organ donation: a critical review and synthesis of individual and next-of-kin donation decisions. Health Psychol. 16, 183–195. 10.1037/0278-6133.16.2.183 9269891

[B41] Ralph A. Chapman J. R. Gillis J. Craig J. C. Butow P. Howard K.. (2014). Family perspectives on deceased organ donation: thematic synthesis of qualitative studies. Am. J. Transplant. 14, 923–935. 10.1111/ajt.12660 24612855

[B42] Robbins M. L. Levesque D. A. Redding C. A. Johnson J. L. Prochaska J. O. Rohr M. S.. (2001). Assessing family members' motivational readiness and decision making for consenting to cadaveric organ donation. J. Health Psychol. 6, 523–535. 10.1177/135910530100600506 22049451

[B43] Rodrigue J. R. Cornell D. L. Howard R. J. (2006). Organ donation decision: comparison of donor and nondonor families. Am. J. Transplant. 6, 190–198. 10.1111/j.1600-6143.2005.01130.x 16433774 PMC2365918

[B44] Rodrigue J. R. Cornell D. L. Howard R. J. (2008). Does family disagreement affect donation decisions by next of kin? Prog. Transplant. 18, 179–184. 10.1177/152692480801800306 18831483

[B45] Rosenblum A. M. Horvat L. D. Siminoff L. A. Prakash V. Beitel J. Garg A. X. (2012). The authority of next-of-kin in explicit and presumed consent systems for deceased organ donation: an analysis of 54 nations. Nephrol. Dial. Transplant. 27, 2533–2546. 10.1093/ndt/gfr619 22121233 PMC3363979

[B46] Scandroglio B. Domínguez-Gil B. López J. S. Valentín M. O. Martín M. J. Coll E.. (2011). Analysis of the attitudes and motivations of the Spanish population towards organ donation after death. Transplant Int. 24, 158–166. 10.1111/j.1432-2277.2010.01174.x 20964724

[B47] Schaub F. Fischer-Fröhlich C. Wolf C. Kirste G. (2013). Gespräche mit angehörigen zur organspende – retrospektive analyse von 6617 gesprächen [Family approach – retrospective analysis of 6617 donation requests]. Dtsch. Med. Wochenschr. 1380, 2189–2194. 10.1055/s-0033-1349554 24048699

[B48] Shemie S. D. Hornby L. Baker A. Teitelbaum J. Torrance S. Young K.. (2014). International guideline development for the determination of death. Intensive Care Med. 40, 788–797. 10.1007/s00134-014-3242-7 24664151 PMC4028548

[B49] Sherman J. W. Gawronski B. Trope Y. (eds.). (2014). Dual-Process Theories of the Social Mind. New York, NY: Guilford Publications.

[B50] Siminoff L. A. Agyemang A. A. Traino H. M. (2013). Consent to organ donation: a review. Prog. Transplant. 23, 99–104. 10.7182/pit2013801 23448829 PMC6776471

[B51] Siminoff L. A. Alolod G. P. Wilson-Genderson M. Yuen E. Y. Traino H. M. (2017). A comparison of request process and outcomes in donation after cardiac death and donation after brain death: results from a national study. Am. J. Transplant. 17, 1278–1285. 10.1111/ajt.14084 27753206 PMC5395358

[B52] Siminoff L. A. Lawrence R. H. Zhang A. (2002). Decoupling: what is it and does it really help increase consent to organ donation? Prog. Transplant. 12, 52–60. 10.1177/152692480201200110 11993071

[B53] Simpkin A. L. Robertson L. C. Barber V. S. Young J. D. (2009). Modifiable factors influencing relatives' decision to offer organ donation: systematic review. BMJ 338:b991. 10.1136/bmj.b991 19383730 PMC2671586

[B54] Sque M. Long T. Payne S. (2005). Organ donation: key factors influencing families' decision-making. Transplant. Proc. 37, 543–546. 10.1016/j.transproceed.2004.11.038 15848450

[B55] Sque M. Payne S. A. (1996). Dissonant loss: the experiences of donor relatives. Soc. Sci. Med. 43, 1359–1370. 10.1016/0277-9536(96)00002-0 8913005

[B56] Sque M. Payne S. Macleod Clark J. (2006). Gift of life or sacrifice?: key discourses to understanding organ donor families' decision-making. Mortality 11, 117–132. 10.1080/13576270600615260

[B57] Starcke K. Brand M. (2012). Decision making under stress: a selective review. Neurosci. Biobehav. Rev. 36, 1228–1248. 10.1016/j.neubiorev.2012.02.003 22342781

[B58] Staub E. (2003). The Psychology of Good and Evil: Why Children, Adults, and Groups Help and Harm Others. Cambridge: Cambridge University Press.

[B59] Staub E. (2005). The roots of goodness: the fulfillment of basic human needs and the development of caring, helping and nonaggression, inclusive caring, moral courage, active bystandership, and altruism born of suffering, in Moral Motivation Through the Life Span, eds Carlo G. Edwards C. (Lincoln, NE: University of Nebraska Press), 33–72. 16335738

[B60] Steed L. G. Wager W. L. (1998). The bereavement process in organ and tissue donor families. Aust. Psychol. 33, 101–104. 10.1080/00050069808257389

[B61] Stroebe M. Schut H. (2010). The dual process model of coping with bereavement: a decade on. OMEGA J. Death Dying 61, 273–289. 10.2190/OM.61.4.b 21058610

[B62] Stroebe M. S. Hansson R. O. Stroebe W. E. Schut H. E. (2001). Handbook of Bereavement Research: Consequences, Coping, and Care. Washington, DC: American Psychological Association.

[B63] UNOS (2017). United Network for Organ Sharing DSA Dashboard – Comprehensive Data Through September 2016. Available online at: http://www.aopo.org/related-links-data-on-donation-and-transplantation (Accessed January 30, 2017).

[B64] van Doorn J. Zeelenberg M. Breugelmans S. M. (2014). Anger and prosocial behavior. Emot. Rev. 6, 261–268. 10.1177/1754073914523794

[B65] van Leiden H. A. Jansen N. E. Haase-Kromwijk B. J. (2010). Higher refusal rates for organ donation among older potential donors in the Netherlands: impact of the donor register and relatives. Transplantation 90:677. 10.1097/TP.0b013e3181eb40fe 20606603

[B66] Vollhardt J. R. (2009). Altruism born of suffering and prosocial behavior following adverse life events: A review and conceptualization. Soc. Justice Res. 22, 53–97. 10.1007/s11211-009-0088-1

[B67] Walker J. A. McGrath P. J. MacDonald N. E. Wells G. Petrusic W. Nolan B. E. (1990). Parental attitudes toward pediatric organ donation: a survey. Can. Med. Assoc. J. 142, 1383–1387. 2350757 PMC1451975

[B68] Walker W. Broderick A. Sque M. (2013). Factors influencing bereaved families' decisions about organ donation: an integrative literature review. West. J. Nurs. Res. 35, 1339–1359. 10.1177/0193945913484987 23618820

[B69] Weiss J. Coslovsky M. Keel I. Immer F. F. Jüni P. (2014). Organ donation in Switzerland-an analysis of factors associated with consent rate. PLoS ONE 9:e106845. 10.1371/journal.pone.0106845 25208215 PMC4160222

